# Effects of low carbohydrate diets high in red meats or poultry, fish and shellfish on plasma lipids and weight loss

**DOI:** 10.1186/1743-7075-4-23

**Published:** 2007-10-31

**Authors:** Bridget A Cassady, Nicole L Charboneau, Emily E Brys, Kristin A Crouse, Donald C Beitz, Ted Wilson

**Affiliations:** 1 Department of Biology, Winona State University, Winona, MN, USA; 2 Department of Animal Science, Iowa State University, Ames, IA, USA

## Abstract

**Background:**

Low carbohydrate diets (LCDs) have been demonstrated to be effective tools for promoting weight loss and an improved plasma lipid profile. Such diets are often associated with increased meat consumption, either poultry, fish, and shellfish (PFS), which are generally high in polyunsaturated fat (PUFA) or red meats (RM), generally high in saturated fat (SFA). The fatty acid profile and content of a diet may influence the plasma lipid profile of humans. This study examined whether the type of meat consumed could influence the outcome of an LCD.

**Methods:**

Moderately obese subjects consumed two different LCDs as part of a weight loss regimen: 1) a diet high in foods of mammalian origin (RM) intended to contain more SFA, or 2) a diet high in PFS intended to contain more PUFA. Diet dependent changes in body weight, nutritional intake, and plasma lipids were evaluated during a 28 day study period.

**Results:**

Both diets were associated with significant weight loss after 28 days, -5.26 ± 0.84 kg and -5.74 ± 0.63 kg for RM and PFS groups, respectively. The PFS diet was associated with a significantly higher intake of PUFA and cholesterol. Despite high cholesterol and fat intakes, neither diet was associated with significant changes in plasma cholesterol or the plasma lipoprotein cholesterol profile. While plasma triglycerides were reduced in both groups, the effect was only statistically significant for the PFS diet.

## Background

Obesity is a primary risk factor for coronary heart disease (CHD) and mortality [1]. Between 2001 and 2002, 65.7 % of Americans were classified as clinically overweight or obese [2]. Presently, 29% of men and 24% of women claim to be attempting to lose weight or maintain previously achieved weight losses [3]. Orthodox dietary recommendations seek to promote weight loss, weight control or improved plasma lipid profiles by placing upper limits on the total intake of sugars, fat, and cholesterol [4,5]. The American Heart Association, for example, recommends that persons consume less than 300 mg cholesterol/day [4]. A fat intake of greater than 35 % of total calories has been suggested to increase saturated fat intake which, in theory, also makes avoidance of excess caloric intake difficult [5]. The same guidelines suggest maintaining carbohydrate intake between 45 and 65% of total calories [5].

Low carbohydrate diets (LCD) are associated with a fat intake percentage that typically exceeds orthodox recommendations. LCDs represent a way to reduce caloric intake, promote weight loss, and reduce the atherogenicity of the plasma lipid profile. The meta-analysis of studies between January 1, 1980 and February 28, 2005 by Nordmann et al [6] demonstrated that relative to low-fat energy restricted diets, LCDs have a better efficacy for weight loss promotion and improved plasma lipid profiles than low-fat diets during the first six months of administration [6]. LCDs have been demonstrated to lead to significant improvements in the plasma lipid profile and, presumably, CHD risk [7-10]. The benefits of LCDs on the lipid profile have been shown to occur independently of weight loss [9,11].

LCDs are often referred to as 'ketogenic' because of the accumulation of ketone bodies in the plasma and urine that is observed when carbohydrate restriction is imposed. Effective weight loss with a calorie-restricted LCD does not absolutely require the establishment of ketosis, although detection of ketosis is often used as a biomarker for monitoring dietary compliance [12,13].

Increased cholesterol and saturated fatty acid (SFA) intake has been associated with increased CHD mortality [14]. In contrast, others have suggested that SFA limitations may be unwarranted, especially when considered in the context of a LCD [15]. Palmitic acid is a primary dietary SFA and tends to be hypercholesterolemic [16], though not if administered in the presence of linolenic acid [17]. Stearic acid is primarily obtained from beef fat [18] and increased stearic acid intake actually improves the lipid profile [16,18]. Diets high in polyunsaturated fatty acids (PUFA) of marine origin tend to decrease plasma triglycerides, improve the plasma cholesterol profile, and reduce the risk of CHD [18-20]. Fish and fish oils are primary sources of eicosapentaenoic acid and docosahexaenoic acid, the two primary protective n-3 oils [21]. These associations lead to the promotion of increased fish consumption as part of the American Heart Association's dietary recommendations for reducing CHD [4].

The content of SFA and PUFA in different foods varies greatly [22]. Food products derived from mammals (e.g. milk, cheese, and meats) tend to contain SFA to PUFA ratios of 11:1 to 4:1. Food products derived from poultry (especially eggs), fish (mainly cold water varieties) and shellfish (including shrimp) tend to have SFA to PUFA ratios of 3:1 or less. These two different food groupings have the potential to exert different effects on plasma triglycerides and lipoprotein cholesterol. Previous studies of the LCD have not compared different types of foods with different lipid profiles for effects on weight loss and the plasma lipid profile.

The present study examined the dietary effects of two different diets under free-living conditions encountered by typical dieters using an LCD. The LCD diets compared in this study were either high in foods of mammalian origin (red meats; RM) and presumed to be high in SFA, or high in poultry, fish, and shellfish (PFS) and presumed to be high in PUFA.

## Methods

### Subjects and design

Subjects were recruited for the study following ethical approval by the Winona State University Human Subjects Committee. Subjects were self-described as overweight, non-smokers between the ages of 30 and 50 years who were not currently taking cholesterol-lowering drugs. Subjects were required to refrain from alcohol for one week prior to the start of the study (January 10, 2005) and for the remainder of the 28 days of the study. Subjects were screened for eligibility by phone and 21 eligible subjects were invited to attend an orientation session during the week prior to the study. Eighteen individuals, 6 males and 12 females, participated in the study. The baseline body mass index of the 12 moderately obese persons who completed the study was 31.71 ± 0.97 (kg/m^2^). The primary motivation for study participation was achievement of desired weight loss. Subjects received no monetary compensation for their participation.

This study sought to determine the effect of a LCD composed of either RM (red meats or products of mammalian origin, and presumably containing more SFA) or PFS (poultry, fish and shellfish, and presumably containing more PUFA) on weight loss and plasma lipids. The study attempted to create conditions that would be similar to those encountered by typical free-living people attempting to lose weight in a non-clinical atmosphere.

At an orientation session, subjects were familiarized with the mechanics and goals of a LCD regimen. As a target, individuals were encouraged to consume a maximum of 20 g of carbohydrates per day that provided 1,487 total calories (Kcals), with 7 % of calories from carbohydrates, 43% of calories from protein, and 50% from dietary fat [23]. Subjects were provided with a review outlining meats deemed as acceptable in a RM (meats or meat products of mammalian origin, excluding those of PFS origin) or PFS (excluding those of RM origin) LCD diet. Subjects were instructed to consume on a daily basis not more than three cups of salad greens or two cups of salad greens and a cup of cooked low-glycemic index vegetables similar to those previously described [24]. Subjects were also asked to exclude caffeinated beverages and other sweetened beverages from their diet. Alcohol intake was limited to no more than one drink per week. Foods high in soy protein were permitted for either group, although diet records revealed soy-products were seldom consumed by either group. Since PFS subjects expressed concern about being able or willing to avoid cheese (mammalian origin and high in saturated fat) for 28 days, no more than 3 oz of cheese per day was permitted for both groups as a way to improve dietary compliance. However, cow's milk was not permitted by either group. The subjects were randomly assigned to a LCD high in RM or PFS and for the following 28 days and the subjects of both groups were responsible for purchasing, preparing, and consuming the foods specific to their assigned group. At the instructional session, subjects were also shown how to maintain a food journal that would record daily food intake during the study.

Subjects were contacted by phone or e-mail one to three times per week in order to answer any questions about dietary compliance, food journal completion, and to promote improved dietary compliance. Aerobic exercise was suggested as a means of promoting maximal weight loss, but no attempt was made to quantify subject physical activity during the study.

Following an overnight fast (≥ 9 hours; water only), subjects visited the laboratory between 6:00 and 8:00 AM on days 0, 7, 14, 21, and 28. During these visits subjects submitted their food journals, were weighed, and provided with a new food journal for self-recording of daily food intake. Journals were used for monitoring dietary compliance and to provide subjects with feedback on what foods were and were not acceptable. After the interview, urine samples were collected and analyzed for the presence of ketones with Bayer Multistix 10 SG reagent strips. Urine samples were tested immediately to determine if subjects had reached a ketotic state. The presence of > 5 mg of ketones/dL was considered positive.

### Plasma lipid analysis

Venous blood samples were collected on days 0 and 28 and centrifuged to obtain plasma which was evacuated with nitrogen gas, flash frozen in liquid nitrogen, and stored at -80°Celsius until analysis. At the conclusion of the study, plasma total cholesterol, LDL cholesterol, HDL cholesterol, and triglyceride concentrations were measured in duplicate by using a Dade Behring Dimension RxL clinical chemistry analyzer and direct measurement (Deerfield, IL). The plasma fatty acid profile of day-28 plasma samples was generated to assess overall dietary compliance. Plasma was extracted using a modified Folch wet tissue lipid extraction [25]. Total lipids were esterified using acetyl chloride/methanol for 1 hour at 80°C [26]. A temperature-programmed procedure was used [27] and fatty acids were identified by comparing the retention time with the GLC 461 standard purchased from Nu-chek-Prep (Elysian, MN). The fatty acid composition was calculated using the peak areas on a percentage basis. Eleven plasma samples (RM = 4; PFS = 7) were found to be suitable for plasma fatty acid analysis.

### Nutritional analysis

Nutritional content of the two diets was analyzed using the weekly food diaries and the Interactive Healthy Eating Index (IHEI) dietary assessment tool [28]. Nutrient intake profiles for the 28-day study were created for each subject and the average nutrient intake between day 0 and 28 was calculated for each subject.

### Statistical analysis

Collected data were analyzed by using the Microsoft Excel data analysis package for descriptive statistics and two-tailed Student's *t*-test for comparison between the two treatment groups (Microsoft Corporation, Redmond, WA). The Statistical Analysis Software (SAS) Program (8.0) was used to perform a repeated measures analysis for evaluating the significance of changes in weight across time within each group (SAS Institute, Cary, NC). All descriptive values were expressed as mean ± standard error of the mean (SEM).

## Results

### Subject attrition

Of the 18 persons who started the study (RM; n = 9 and PFS; n = 9), data collected from 12 subjects (RM; n = 5 and PFS; n = 7) were used in the final analysis. Data were not used from six subjects: one dropped out voluntarily, one was dropped when found to have been taking Lipitor for the control of blood lipids throughout the study, one was dropped due to taking large dose NSAIDs for an injury, two were dropped from the study because of gross dietary non-compliance, and one was dropped for failure to provide a detailed dietary record.

### Effect of diet on weight and ketones

**Figure 1:**
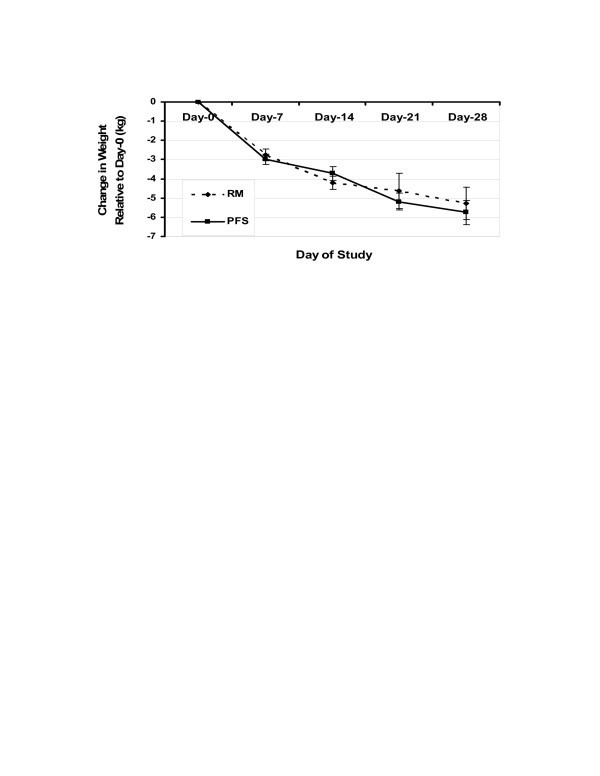
Body weight changes relative to starting weight during the consumption of a low-carbohydrate diets high in either red meat (RM) or poultry/fish/shellfish (PFS) for 28 days. No significant differences between groups were observed at any given week, but there was a statistically significant effect by time P < 0.001.

Baseline body mass index (kg/m^2^) in the RM and PFS groups was 32.72 ± 1.12 and 30.99 ± 1.50, respectively; there was no significant difference between the groups. After consumption of the diets for 28 days, the RM and PFS group lost -5.26 ± 0.84 kg and -5.74 ± 0.63 kg, respectively (Fig 1). Weight loss was similar and statistically significant within both groups across time (p < 0.001), but there was no significant difference between the groups at any given time. Urinary ketones were detected at or above 5 mg/dl on 75% of all test times on days 7, 14, 21, and 28 (Table 1).

**Table 1:** Absence or presence of urinary ketones (above 5 mg/dl) in urine samples for subjects consuming a low-carbohydrate diet for 28 day^1^

Treatment	Ketones	Day 0	Day 7	Day 14	Day 21	Day 28
RM	Absent	5	1	1	1	1
	Present	0	4	4	4	4
PFS	Absent	7	2	2	2	2
	Present	0	5	5	5	5

^1^Mean ± SEM; RM, red meat (n = 5); PFS, poultry/fish/shellfish (n = 7)

### Effect of diets on nutrient intake

Average carbohydrate intake of the RM and PFS groups was not significantly different at 61 ± 27 and 50 ± 22 g/day, respectively. The intake of total calories, protein, fiber, total fat, SFA, and monounsaturated fat was also not significantly different between the two groups over the course of the 28-day study period (Table 2), although SFA intake did approach statistical significance. However, persons consuming the PFS diet did consume significantly more PUFA (p = 0.031) and cholesterol (p = 0.036) than persons consuming the RM diet.

**Table 2:** Average nutrient intake during the consumption of low-carbohydrate diets high in red meat or poultry/fish and shellfish for 28 days^1^

Diet Type	Carb (g/day)	Carb (% cal)	Protein (g/day)	Protein (% cal)	Total Fat (g/day)	Total Fat (% cal)
RM	62 ± 28	17 ± 3.6	106 ± 9	31 ± 1.7	82 ± 10	52 ± 2.7
PFS	50 ± 22	18 ± 2.5	101 ± 5	30 ± 1.0	77 ± 3.7	52 ± 2.4

	Total Calories	Chol (mg/day)	SFA (g/day)	PUFA (g/day)	MUFA (g/day)	Fiber (g/day)

RM	1410 ± 155	333 ± 39^2^	32 ± 5^3^	9.8 ± 2.0^4^	32 ± 4	6.4 ± 1.0
PFS	1342 ± 30	630 ± 95	25 ± 2.0	16 ± 1.5	30 ± 1.8	6.5 ± 0.8

^1^RM, Red meat (RM; n = 5); Poultry/fish/shellfish (PFS; n = 7) Carbohydrates (Carb); Cholesterol (Chol); Saturated fat (SFA); Polyunsaturated fat (PUFA); Monounsaturated fat. Data expressed as mean ± SEM;

^2^Statistically significant difference between treatments (p = 0.031)

^3^Approached statistically significant difference between treatments (p = 0.138)

^4^Statistically significant difference between treatments (p = 0.036)

### Effect of diets on plasma fatty acids

**Figure 2:**
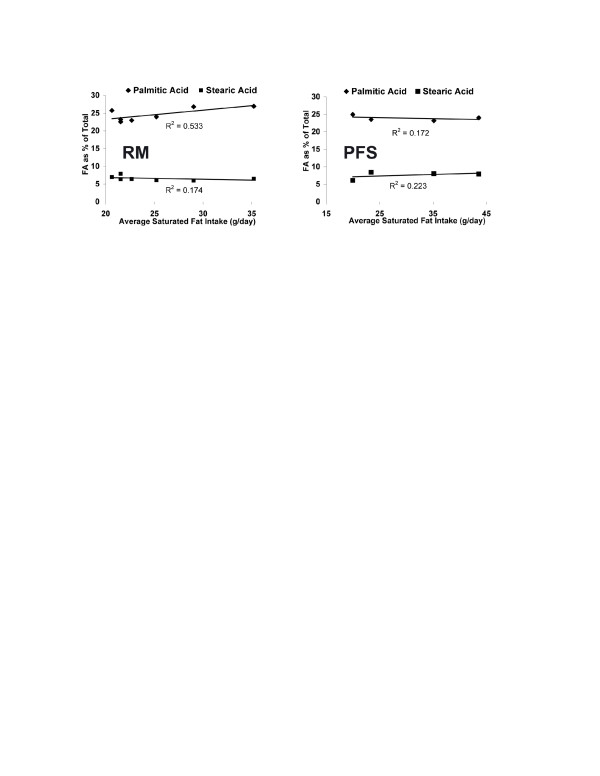
Plasma levels of palmitic and stearic acid were not correlated with saturated fat intake as part of a 28 day low carbohydrate diet rich in red meat (RM) or poultry/fish/shellfish (PFS).

As a percentage of total plasma fatty acids, the consumption of a RM and PFS diet for 28 days was associated with differences in the plasma fatty acid profile (Table 3) A statistically significant difference between the two groups was observed for docosapenaenoic acid (DPA; p = 0.051). A near significant difference between groups was observed for stearic acid (p = 0.071), eicosapentaenoic acid (EPA; p = 0.170) and docosahexaenoic acid (DHA; p = 0.095). The average SFA intake for each person during the 28 day study in the RM and PFS groups was not correlated with plasma palmitic and stearic acid, expressed as a percentage of total plasma fatty acids (Figure 2).

**Table 3:** Plasma fatty acid profile (percentage of total) following the consumption of a low-carbohydrate diet for 28 days^1^

	Percent of Total Fatty Acids	
	
	RM	PFS
Myristic (C14:0)	0.55 ± 0.03	0.74 ± 0.13
Palmitic (C16:0)	23.92 ± 0.38	24.95 ± 0.67
Palmitoleic (C16:1)	1.35 ± 0.15	1.35 ± 0.11
Stearic (C18:0)	7.63 ± 0.52^2^	6.65 ± 0.26
Oleic (C:18:1; n9)	19.83 ± 1.19	20.60 ± 1.75
Linoleic (C18:2; n6)	32.32 ± 1.73	31.80 ± 2.09
Linolenic (C18:3; n6)	0.83 ± 0.50	0.40 ± 0.04
Behenic (C22:0)	0.42 ± 0.22	0.33 ± 0.11
Homogamma Linolenic (C20:3; n6)	0.81 ± 0.25	0.70 ± 0.05
Arachidonic (C20:4; n6)	10.30 ± 1.52	10.15 ± 0.72
Eicosapentaenoic (C20:5; n3)	0.34 ± 0.06^3^	0.57 ± 0.11
Docosapentaenoic (C22:5; n3)	0.48 ± 0.08^4^	0.31 ± 0.01
Docosahexaenoic (C22:6; n3)	1.29 ± 0.18^5^	1.71 ± 0.13
Total n3	2.11 ± 0.31	2.49 ± 0.26
Total n6	44.27 ± 1.01	43.05 ± 1.89

^1^RM, Red meat (RM; n = 5); Poultry/fish/shellfish (PFS; n = 7) Carbohydrates (Carb); Cholesterol (Chol); Saturated fat (SFA); Polyunsaturated fat (PUFA); Monounsaturated fat. Data expressed as mean ± SEM;

^2^Statistically significant difference between treatments (p = 0.031)

^3^Approached statistically significant difference between treatments (p = 0.138)

^4^Statistically significant difference between treatments (p = 0.036)

### Effect of diet on plasma triglycerides and lipoproteins

Consumption of PFS for 28 days was associated with a significant (p = 0.042) reduction in plasma triglycerides relative to day 0 and a non-significant decline in plasma TG for RM consumers (p = 0.326) (Table 4). The changes in plasma total, LDL, and HDL cholesterol between day 0 and 28 were non-significant.

**Table 4:** Change in the plasma triglyceride and lipoprotein concentration following the consumption of a low-carbohydrate diet for 28 days^1^

Day	Triglycerides (mmol/L)	Total Cholesterol (mmol/L)	LDL Cholesterol (mmol/L)	HDL Cholesterol (mmol/L)
RM-0	1.04 ± 0.17	4.78 ± 0.35	2.84 ± 0.26	1.50 ± 0.13
RM-28	0.83 ± 0.11	4.47 ± 0.34	2.79 ± 0.22	1.31 ± 0.12
RM-Change	-0.21 ± 0.19	-0.30 ± 0.15	-0.05 ± 0.14	-0.19 ± 0.05
PFS-0	1.27 ± 0.13	5.10 ± 0.31	3.18 ± 0.35	1.30 ± 0.14
PFS-28	0.87 ± 0.08^2^	4.95 ± 0.31	3.33 ± 0.27	1.13 ± 0.10
PFS-Change	-0.39 ± 0.10	-0.15 ± 0.23	0.15 ± 0.21	-0.17 ± 0.08

^1^RM, red meat; PFS, poultry/fish/shellfish; Mean ± SEM

^2^Statistically significant difference for triglycerides within PFS treatment between day 0 and 28 (p = 0.042)

## Discussion

In this 28-day study of moderately obese persons, both the RM and PFS LCD effectively promoted significant weight loss (Table 1). Caloric intake of the two groups differed by 68 Kcal/day. No significant difference in total weight loss was observed between the RM and PFS groups, -5.26 ± 0.84 and -5.74 ± 0.63 kg, respectively. Weight loss experienced by the RM and PFS groups in the current study was comparable to that observed on week four of other previous LCD studies [12,13,29,30].

The carbohydrate intake for both the RM and PFS groups was 17 ± 3.6 and 18 ± 2.5% of total calories, respectively, and significantly lower than the current USDA recommendation of 45–65% [5]. The degree of carbohydrate restriction in the current study was similar to some previous studies [9,12] but greater than others [7,8,10,13,30] and greater than the target value of the Atkins induction phase [24].

Persons consuming the PFS diet had a significantly greater PUFA intake than those in the RM group (p = 0.036), although the difference in specific n-3 and n-6 PUFAs for the diets was not calculated. The SFA intake of the RM group was slightly elevated, relative to the PFS group. Persons in the RM group had a mean intake of SFA that was 32.42 ± 4.62 g/day and the SFA intake of the PFS group was 25.12 ± 2.01 g/day, this difference approached significance (p = 0.137). In interviews with subjects prior to the start of the study, it was determined that cheese was a major element that subjects in both the RM and PFS groups strongly preferred to include in their diets. The investigators chose to permit controlled and documented cheese consumption in the PFS group in order to improve honest dietary reporting and compliance. In light of the high SFA content of cheese, it was not surprising that the difference between the two groups for SFA intake only approached significance.

The day 28 plasma fatty acid profile of EPA, DHA, and DPA suggests that the RM and PFS groups were compliant with regards to consuming their assigned diets (Table 3). Two specific PUFAs that are indicative of PFS consumption are the n-3 marine oils EPA and DHA [21,31]. Plasma fatty acid levels of EPA and DHA in the PFS group were both greater than that observed in the RM group. The differences approached significance with p-values of 0.170 and 0.095 for EPA and DHA, respectively. Subjects consuming the RM diet, which typically contained beef products, would be expected to consume more of the non-marine n-3 fatty acid DPA [32] and stearic acid [16,18]. The group-specific difference for DPA was significant (p = 0.051) and approached significance for stearic acid (p = 0.071). Figure 2 demonstrates that within the RM and PFS groups, when expressed as a percentage of the total, plasma palmitic or stearic acid was not tightly correlated with total SFA intake, suggesting that the control of the plasma levels of these two fatty acids is under predominately metabolic, not dietary control.

The PFS diet resulted in the consumption of nearly twice the cholesterol (630 ± 95 mg/day) as members of the RM group (333 ± 39 mg/day). This high cholesterol intake was mostly attributable to shrimp and eggs consumed on 25 and 88% of all days, respectively. Subjects made these cholesterol-rich foods a staple of their self-purchased free-living diets because of taste preferences, familiarity, and product availability. Studies of human egg and shellfish consumption have suggested that cholesterol content has, at best, only a small impact on the plasma cholesterol profile [33-35]. Consumption of large amounts of cholesterol-rich eggs and shrimp by PFS subjects may have influenced the expected improvements in the plasma lipid profile that would be expected to accompany the observed weight loss [9,36]. The moderate degree of carbohydrate restriction observed in the current study may have been inadequate to produce the LCD induced changes in the plasma cholesterol profile associated with the previously described studies.

Subjects experienced a significant reduction in plasma triglycerides in the PFS group (p = 0.042) while plasma triglyceride reduction in the RM group was not significant (p = 0.326). Because the RM subjects began the study with a plasma TG concentration that was generally lower than that of the PFS group they may have been less responsive to diet induced reductions in plasma triglycerides.

The total daily fiber intake recommendation of the Food and Nutrition Board is 38 and 25 g/day for men and women [37]. In the current study, fiber intake for RM and PFS subjects was 6.4 ± 1.1 and 6.5 ± 0.8 g fiber/day, respectively. It has been previously demonstrated that increased fiber intake as part of a LCD may improve potential reductions in LDL cholesterol, though it is not required for improvements in the plasma lipid profile [10].

## Conclusion

This study determined that a 28 day LCD high in RM or PFS promotes a similar significant degree of weight loss. In spite of orthodox concerns about the impact of dietary cholesterol and saturated fat, no significant deleterious changes were observed to result from adherence to either the RM or PFS diets, which were both high in total cholesterol and total fat. This study suggests that dire predictions about LCD effects are anachronistic.

## Competing interests

The author(s) declare that they have no competing interests.

## Authors' contributions

BAC and NLC analyzed subject dietary records for nutritional intake data, performed statistical analysis of data, and prepared the initial manuscript. EEB and KAC recruited/organized subjects, collected physiological data, and prepared/handled blood samples. DCB supervised the plasma fatty acid analysis. TW designed and organized the study. BAC and TW prepared the final manuscript which was read and approved by all authors.
